# Meetings of Societies

**Published:** 1896-06

**Authors:** 


### MEETINGS OF SOCIETIES.

### Bristol Medico-Chirurgical Society.

*March 11th*, 1896.

Mr. Arthur W. Prichard, President, in the Chair.

Dr. F. H. Edgeworth showed two patients. The first was a man, aged 29, suffering from a neuritis of the median nerve of the right arm, resulting in wasting, some loss of power and reaction of degeneration in the muscles innervated, and partial anæsthesia in the skin area supplied. The nerve was tender on pressure both at the elbow and wrist. The interest of the case lay in its etiology. The man is by trade a bootmaker, and was in good health until four months ago, when he for the first time began to work with a machine called an "edge setter," which consists of a rapidly vibrating block of steel against which the edge of the sole of a boot or shoe is held to smooth and polish it. The vibration is felt most disagreeably throughout the whole body, though more especially in the right forearm. Soon after working at this machine he began to experience a weakness in the right arm, which gradually increased so that in about a month he had to give up this branch of his trade. His arm then got better, but again became weaker when two months later he was obliged to use the machine once more. No other cause for the neuritis could be found. The patient says that men who begin to work with this edge setter are apt to experience a temporary weakness in the right arm; but he does not know any case so well marked as his own. It seems fairly clear then that the neuritis is of traumatic origin, set up by the vibrations of the machine. The condition had considerably improved with massage. The second case was one of ataxic paraplegia in a man aged 43. The disease was of slow origin and of about five and a half years' duration. There was a spastic paraplegia of the legs, evidenced by weakness, spastic gait, and exaggerated knee-jerks, with rectus and ankle-clonus on both sides, though a little more marked on the left. In addition there was well-marked ataxy. The bladder and rectum were slightly affected. The disease was to be differentiated from locomotor ataxy by the exaggerated knee-jerks, absence of lightning pains, and normal pupillary reactions, and there was no history or sign of syphilis; whilst it was unlike spastic paraplegia in that ataxy was present. Myelitis was excluded by the chronic and progressive course.—Dr. Watson Williams, commenting on Dr. Edgeworth's case of ataxic paraplegia with exaggerated knee-jerk, referred to two recently recorded cases in which the patellar tendon-reflexes reappeared owing to the onset of descending degeneration in the lateral tracts; viz.: a case of locomotor ataxia, under Raichline, in which, with the onset of left hemiplegia with secondary contracture, the left knee-jerk reappeared; the other instance being that of a diabetic patient, observed by Marinesco, in whom Weber's syndrome (superior crossed hemiplegia) supervened, with the reappearance of the lost knee-jerk on the affected side.—Dr. Henry Waldo asked Dr. Edgeworth if he had any experience of the hypodermic injection of strychnine in cases of peripheral neuritis. It had been much praised by Grainger Stewart, and Dr. Waldo had found it valuable in some cases.—Mr. C. A. MORTON asked why, supposing the neuritis was due to the vibrations, the median nerve only had suffered.—Dr. MICHELL CLARKE said that the ataxic patient had been under his care for some time previously, and that he agreed with Dr. Edgeworth that the case was one of ataxic paraplegia. The symptoms were practically the same two years ago as now; the ataxy was then relieved for a time by suspension.—In reply Dr. EDGEWORTH said that in reference to the case of neuritis, he could offer no explanation why the median nerve alone was affected. The hypodermic use of strychnine was not indicated in this case, as there was much local tenderness and inflammation, and improvement was considerable under the treatment adopted. The first case of locomotor ataxy with absent knee-jerks in which a hemiplegia had brought back the knee-jerk had been, he believed, recorded by Hughlings Jackson and Taylor. The existence of a knee-jerk in a person suffering from ataxic paraplegia showed that the sclerosis of the posterior columns could not be complete; for, if complete, the muscle reflex arc would be broken, and no amount of lateral sclerosis would bring it back.

Dr. BERTRAM ROGERS showed an anencephalous monster which had been sent to the Children's Hospital by Mr. John Griffiths, in whose practice the case occurred. The woman was a primipara (as is usually the case), and went to the seventh month before the confinement came on. The fœtus presented by the head, and was born without difficulty—the mother making a good recovery. No maternal impressions are alleged to have caused the monster. The fœtus is without brain, and only a small string of spinal cord is apparent down the open spinal column; and it presented the other usual features of these monsters, such as absence of neck and exophthalmos.—Dr. JOHN BROOM related a case which had occurred in his practice. The labour was at full term and was natural. There was neither cerebrum nor cerebellum, the skull being lined only by an extension of the dura mater from the medulla oblongata which terminated just within the foramen magnum. —Dr. J. G. SWAYNE said that the anencephalous fœtus shown resembled most specimens he had seen, except that the amount of brain was less developed than usual.

Dr. THEODORE FISHER showed two specimens. The first was one of hæmorrhagic pancreatitis which occurred in a woman, aged 56, who died of hæmorrhage into the right optic thalamus. The pancreas was dotted over with small hæmorrhages, and section showed blood infiltrating the interstitial tissue of the gland, and the lobules of reddish-grey colour, which microscopically showed disintegration of the cells. The kidneys were small and granular, and there was general arteriosclerosis; but the heart was not enlarged. This case had not the clinical interest of most cases of the disease, because it occurred as a complication of cerebral hæmorrhage and was in all probability not the cause of death. The second specimen was one of thrombosis of the superior vena cava above the vena azygos major, and of the right and left innominate veins. The cause of the thrombosis was not obvious. There were some enlarged reddened glands in the mediastinum, and microscopically the mediastinal tissues showed proliferation of the fat-cells and small-celled infiltration. Possibly this inflammation of the mediastinum was primary, and the cause of the thrombosis. —Dr. SHINGLETON SMITH spoke on the clinical aspects of the case from which Dr. Fisher's second specimen was taken. The patient, a middle-aged woman, was admitted with symptoms of obstruction of the superior vena cava; but there were no indications of aneurysm or any other intra-thoracic tumour. The only point of importance in the history of the patient was the fact that there had been eleven miscarriages (with no child born alive), although there was no other evidence of syphilis; whether this thrombosis was primary or dependent upon some chronic mediastinal inflammation seemed difficult to determine.—In reply Dr. FISHER said that there was no evidence that the inflammation of the mediastinum was gummatous. There was very little fibroid thickening and no caseation.

The PRESIDENT showed a six months' fœtus, and the uterus which he had removed from a patient whose abdomen had been penetrated by a revolver bullet. The bullet had entered the child to the right of the spine about the neck of the eighth or ninth rib, and the exit-wound was in the costal cartilages of the same side, the shot thus passing through the pleura, diaphragm, and liver; the placenta also was perforated. The case was fully reported and illustrated in the *British Medical Journal* of February 8th, 1896.—Dr. J. G. SWAYNE said that Mr. Prichard's case was exceedingly interesting from an obstetrical as well as from a surgical point of view. When he began lecturing on Midwifery, more than fifty years ago, Naegele's view as to the relative frequency of the different cranial presentations prevailed almost universally in Great Britain. Dr. Swayne doubted its correctness from the first, and was soon led to disbelieve it in consequence of meeting with a number of cases in which he found the child to lie in the second position, even before labour had commenced. Had a kind of *experimentum crucis* been needed to prove this, a better case than Mr. Prichard's could not have been selected.

Dr. BERTRAM ROGERS gave a demonstration of Soxhlet's milk steriliser. —Dr. SHINGLETON SMITH was not convinced of the advantages of a special process of sterilisation over ordinary boiling. If milk could be obtained fresh and was likely to be consumed within a reasonable time, there was no necessity for sterilisation; but he quite approved of the habitual practice of boiling all milk before use. Ordinary boiling was an efficient method of sterilising, and other more elaborate methods did not appear to have any advantages over it for ordinary every-day use. The coagulation of albumen, and the change in the taste, must occur with any process of sterilisation, just as in ordinary boiling.

Mr. C. W. J. BRASHER recorded "A Case of Ligature and Division of the Vasa Deferentia for Prostatic Hypertrophy" (see p. 150).—Dr. JAMES SWAIN referred to Professor White's statement that atrophy of the prostate ensued after ligature of the spermatic cord as a result of atrophy of the testes, and to the experiments of Griffiths and others which showed that atrophy of the testes and prostate did not usually follow ligature or division of the cord except in a few instances. Experiment, therefore, tended to show that we could not expect atrophy of the prostate from ligature of the spermatic cord. It was not always allowable to argue from dogs and rabbits to human beings: and there had now been reported a fair number of cases in the human being in which prostatic atrophy *had* followed ligature of the spermatic cord. If these cases were further supported by others, we may have to reconsider the aspect of castration as opposed to ligature of the cord for hypertrophy of the prostate. For in favour of the latter operation we have (1) a lessened shock; (2) the retention of organs which may possibly serve other purposes in the economy than fertilisation of the ovum; and (3) it satisfies the æsthetic objections of those who decline to lose their testicles, even though celluloid balls can be supplied to take their place. Mr. Harrison's recently published cases[1] went to show that it was only necessary to tie the vas deferens and not the vascular elements of the cord as well, as Mr. Brasher had done. This too was borne out by the more recent experiments of White,[2] in which atrophy of the prostate had been produced in dogs after division of the vas deferens without any apparent change in the testes. The method of operating adopted by Harrison is simple. He isolates the cord just as it is entering the scrotum, raises the vas deferens in a loop with a blunt hook, and then ties a ligature round the loop, which is afterwards cut away. Dr. Swain thought that this operation might be done, if desirable, under the influence of cocaine without the use of a general anæsthetic.—Mr. C. A. Morton thought that whereas castration for prostatic hypertrophy had passed out of the experimental stage, and could be recommended to patients with confidence in properly selected cases, division of the vas deferens was still only in the experimental stage, and that the evidence as to its value was extremely conflicting. He referred to the observation made by Hunter many years ago, that in a body in which, on dissection, the vas was found obliterated, there was no atrophy of the testis; and to Brissaud's experiments on rabbits, which showed that if after ligature of the vasa deferentia the animals were placed by themselves no atrophy took place in the testes; but if placed with the does, the testes, after first enlarging, atrophied from the pressure of the excessive secretion in the tubes on the epithelium. Mr. Brasher's case was distinctly more successful than some of Mr. Harrison's.—Mr. W. M. Barclay considered that Mr. Brasher's operation had been so far very fairly successful, inasmuch as the diminution in the size of the prostate and the effect on micturition were considerable. He then referred to the experiments of Dr. White on dogs, in which ligature of the vasa was uniformly followed by marked atrophy of the prostate, without, however, causing atrophy of the testicles. Supposing these results to be transferable to the human subject, the absence of testicular atrophy was quite the opposite of a disadvantage. The cases so far recorded of ligature of the vasa are too small for a decision for or against the proceeding; but this case certainly was one in its favour. At any rate, it seems legitimate surgery to give the patient his choice, pointing out the greater certainty of orchectomy, as far as our present knowledge goes; and supposing the operation to fail, removal of the testicles can follow.—Mr. Brasher, in reply, said that ligature of the spermatic arteries seemed in this case to have resulted in the earlier relief of the prostatic congestion.

Mr. C. A. Morton reported "A case of Supra-vaginal Amputation of the Cervix for Carcinoma during the fifth month of Pregnancy." There was a large cauliflower-like growth from the cervix, without any infiltration of the vaginal wall or extension towards the body of the uterus. The disease had been discovered by the patient's medical man in the country a month before; and the woman was sent to Mr. Morton at the Bristol General Hospital for its removal. Dr. Lawrence, who saw the case, considered that delivery could not take place through the obstructed cervix, and that it would be needful either to remove the growth or perform Cæsarian section later on; and both Dr. Lawrence and Mr. Morton had no hesitation in deciding in favour of the former. To remove the pregnant cancerous uterus through the abdomen seemed to them an unnecessarily grave operation to undertake, seeing that had the uterus been empty it was a suitable case for supra-vaginal amputa-

[1] *Lancet*, 1896, i. 473. [2] *Ann. Surg.*, 1895, xxii. i.

tion rather than complete hysterectomy, with its far greater risks; and that even if abortion followed the supra-vaginal amputation, as it seemed to them it inevitably must, yet still the risk was less than extirpation of the pregnant uterus. The operation was performed on December 18th, 1895. The cervix was cut through by means of the galvanic ecraseur after reflection of the bladder and the peritoneum of Douglas's pouch, and ligatures had been applied to the base of the broad ligaments and uterine arteries. Abortion occurred on the fourth day. The patient was discharged, with the stump of the cervix healed, on January 16th.—Mr. Greig Smith ventured to disagree with the opinion that it was the best surgery to perform supra-vaginal amputation in all cases of cancerous cervix associated with pregnancy. On the contrary, he believed that total hysterectomy would, in most cases where the pregnancy was early, be the best treatment. He had personally a strong dislike to the actual cautery for such operations, unless it could be applied as Keith applied it, when it was essentially a process of desiccation. If the uterine arteries were properly secured, which ought to be the first step in cervical amputation, the operation was almost a bloodless one.—In reply, Mr. Morton said that he had not recommended supra-vaginal amputation as a general rule in cancer of the cervix in pregnancy, but only in cases like the one he had reported. The question of complete removal of the pregnant uterus had been carefully considered, and it was felt that it was unnecessary to subject the woman to the far greater risks of such an operation, even though abortion was considered inevitable after supra-vaginal amputation. The result so far had abundantly justified such a view.

---

*April 8th*, 1896.

Mr. Arthur W. Prichard, President, in the Chair.

Dr. Watson Williams invited the attention of the members to the Huxley Memorial Fund. The local committee hoped that intending subscribers would at their earliest convenience forward their donations to Messrs. Stuckey & Co., Clifton, marked "Huxley Memorial."

Dr. Watson Williams exhibited two patients: (1) A child, aged 11, with cerebral spastic diplegia, resulting from injury at birth. The child was of about average intelligence, and the only notable phenomena were the usual spastic symptoms. It was peculiar in that the forearms showed clonic adduction and abduction spasmodic movements when the child attempted to use them, especially when being watched. (2) A man, aged 74, with early intrinsic laryngeal cancer. The patient had complained of hoarseness for three months, and this was the only symptom he had noticed. Examination of the larynx revealed a pinkish fringe of outgrowth along the upper surface and margin of each cord in its posterior half. But the abduction of the cords was very markedly impaired, and this fact, in the absence of any indication of disease in or around the crico-arytenoid joint, sufficed to distinguish the affection from pachydermia laryngis, which in appearance and location the new-growth otherwise very closely resembled. The case was eminently suitable for radical extirpation as far as the disease was concerned, it being completely intrinsic and fairly localised. Dr. Williams had asked Dr. Swain to see the patient with a view to operation; but the age of the patient was so advanced that it was considered inadvisable to submit him to the inevitable dangers involved in a radical removal.

Dr. Williams wished, however, to lay stress on the fact that the only symptom complained of was hoarseness, and that his patient illustrated well the points of importance in connection with early malignant disease of the larynx to which he had called attention in a recent communication.[1]—With reference to the case of malignant disease of the larynx, Dr. James Swain agreed with Dr. Williams's diagnosis, and stated that the power of abduction of the cords was much less than a few weeks ago. When the case was first sent to Dr. Swain the local condition showed it as one eminently fit for operation from a local point of view, for the disease had existed only two months and was entirely confined to the larynx. In such conditions a thyro-chondrotomy and "clearing out" of the thyroid cartilage would remove the disease; but Dr. Swain doubted the desirability of operation in cases which had passed the confines of the larynx. The case was brought before Dr. Swain's colleagues at the Infirmary, and it was thought that, considering the man's age, his chance of life would be longer if he were allowed to go on for the present, with the performance of tracheotomy when required later on. The radical operation was therefore not performed.

Dr. Edward Crossman showed a specimen of perforating duodenal ulcer from a man, aged 44, who on April 3rd had sudden pain at the epigastrium. After apparent improvement, vomiting set in on the following day and continued till the 5th. The abdomen was much distended. There had been no action of the bowels. It was then decided to open the abdomen; but whilst on the road to the hospital the patient died. The necropsy revealed a great quantity of pus and intestinal contents free in the peritoneal cavity. About three-quarters of an inch from the pylorus there was a circular perforation of the anterior duodenal wall about the size of a threepenny-piece. It had rounded edges, and was evidently of some duration.

Mr. Thomas Carwardine read a paper on "A Case of Dermoid of the Neck" (see page 145).—Mr. Munro Smith remarked on the size of the bodies, which he thought was of importance as indicating that they were not morphologically ova. The fact also that in the usual method of formation of ova only one layer (the discus proligerus) is found, and that in these bodies there exists a thick layer of laminated squamous epithelium round each, seems against the theory of ovulation. Mr. Munro Smith was of opinion that these small masses were balls of epithelium formed by the cyst-wall, and triturated by constant movements into a rounded and uniform size.—Dr. James Swain agreed with Mr. Munro Smith that the size of the ovum *was* of distinct morphological importance. Moreover, the cells were of a markedly squamous type in these apparent ova, with the centre consisting of an amorphous structure resembling sebaceous material. Dr. Swain was therefore inclined to regard these rounded bodies as being formed by exfoliation of epithelial cells lining sebaceous follicles, and when moulded therein were "shot out" in the central cavity of the tumour. In this way a large collection of epithelial balls would be collected; and Dr. Swain thought this origin was more likely than their formation by a simple mutual trituration movement, as suggested by Mr. Munro Smith.—Mr. Carwardine, in reply, said that the remark that size had no morphological importance meant that in the presence of facts brought out by considerations of structure and development difference in size had no value. As to the argument that the round bodies could not be homologous with ovarian follicles because they consisted of very many layers instead of one layer of epithelium, it was pointed out that sections of the cyst-wall seemed

[1] *Bristol M.-Chir. J.*, 1896, xiv. 91.

to suggest that the epithelial masses possibly took origin in connection with a single layer of cells. A careful examination of microscopic specimens did not reveal the presence of sebaceous glands in the cyst-wall.

Mr. Paul Bush read notes of "A Case of Compression of the Brain, due to Rupture of the Middle Meningeal Artery," which he had treated by operation, and which ended in complete recovery. The man had fallen on his head from a height of only about six feet. When first seen he had the physical signs and symptoms of fracture of base of the skull; but within half-an-hour of the accident symptoms of compression of the brain commenced and gradually increased. Mr. Bush did not see the patient till three hours after the injury, when he found no indications of fracture of the vault of the skull, but all the usual symptoms and signs of a large clot of blood pressing on the right side of the cerebrum. The patient was then almost comatose; there was bleeding from the nose and ears, there was marked proptosis and fixation of the right eyeball, and the right pupil was dilated and fixed; before the patient lapsed into the comatose condition it was thought that there was paralysis of the left arm and leg. Mr. Bush made a semi-circular flap on the right side, taking in the whole of the skin and muscle of the temporal region: a comminuted fracture was found leading down to the base; some fragments of bone were removed by the forceps, and a further portion taken away by a large trephine; a large firm clot of blood was then removed, leaving a depression in the dura mater the size of the man's fist and situated over the convolutions on either side of the fissure of Sylvius. There was then very free arterial hemorrhage, which appeared to come chiefly from the anterior and lower portion of the depression: this ceased somewhat after a torn anterior branch of the middle meningeal artery had been found and ligatured; but free hemorrhage still continued, and as the patient appeared to be *in extremis*, a large plug of iodoform gauze was pushed down towards the base chiefly—the ends were left outside the wound. The bleeding became very much less at once, and soon ceased entirely; the temporal muscle was sutured with some deep catgut, and the edges of the skin brought together. Three hours after the operation the man was able to recognise his wife, and about five hours after he was able to say a few words. The gauze was removed at the end of eighteen hours; the movements of the right eyeball were impaired for about three weeks, and there was partial facial paresis for a month. The patient, however, made an uninterrupted recovery.

Mr. Munro Smith gave a lantern demonstration of photographs by the X rays, and of cases of chancres on fingers.

Dr. Shingleton Smith and Mr. James Taylor gave a lantern demonstration of some of the ordinary human parasites.

Dr. Michell Clarke and Mr. James Taylor gave a lantern demonstration of nerve cells and fibres stained by Golgi's method.

---

*May 13th*, 1896.

Mr. Arthur W. Prichard, President, in the Chair.

Dr. Shingleton Smith recalled the leading facts of the case of bilateral spasmodic torticollis shown on a previous occasion (see page 100), and again produced the case to show how little was the result which had followed the operation of excision of an inch of both spinal accessory nerves, which was performed by Mr. Harsant on the 1st of April last. Although for a time there was temporary and partial relief, the result of the operation does not justify any further operative interference. The case is an extreme illustration of the difficulties in the treatment of over-active hypertrophied muscles.—Mr. W. H. Harsant said he had been induced to operate from reading the very favourable results which had been obtained by Mr. Noble Smith and others; but in this particular case so many muscles were involved in the spasm, that it was obviously an unfavourable case. As regards the *technique* of the operation, it is generally advised to make the incision as high as possible, beginning at the mastoid process; but in this case the nerve was more readily reached by lifting the anterior border of the sterno-mastoid about three inches from its upper end.—Dr. H. Waldo thought that if the operation had been done earlier there would have been a much better prospect of a good result, the spasm having so spread to other muscles. The over-action of nerve-cells in these cases was generally admitted to affect the lower or spinal cells rather than the cortical cells; so that if the cortical centre could be removed, there would, he thought, be less chance of improvement than with the operation which had been performed. Dr. Waldo was inclined to think that yet more good could be done by a further division of some of the posterior cervical nerve-branches.—Mr. Munro Smith thought that the character of the spasm had altered very considerably since the operation; instead of being distinctly in the sterno-mastoids, it seemed to be now almost entirely in the deep muscles and in the hypertrophied platysma.—Dr. F. H. Edgeworth remarked that the case illustrated the general rule that if in a case of spasmodic torticollis the movements had spread beyond the trapezius and sterno-mastoid, division of the spinal accessory did little good. It was not probable that the movements in the neck were due to the sterno-mastoid, as, unlike the trapezius, that muscle had its nerve-supply solely from the spinal accessory.

Mr. C. A. Morton showed a girl, aged 18, who had suffered from wryneck since three years of age. The deformity had deen due to contraction of the right sterno-mastoid. He had divided by the open method the muscle and several bands of the deep cervical fascia lying just over the large vessels. The result was very satisfactory. It would have been impossible to have divided these bands of fascia safely by the subcutaneous method, which has considerable risk of hemorrhage from injury to a large vessel beneath, even when the muscle alone is divided; and by an aseptic operation the wound healed just as a subcutaneous one would. The condition of the face was interesting, as it showed—what is described as present in some of these long-standing cases—the condition of slight ill-development on the side, and which had been drawn down for fifteen years.

Dr. F. H. Edgeworth showed a girl, aged 6 years, with right hemiplegia and aphasia of seven months duration. The onset was sudden and accompanied by loss of consciousness, but without fever, pain in the head, or convulsions. Very slight recovery had taken place in the arm, more in the face and leg, and there were athetoid movements in the arm. There was no loss of sensation. The tendon-jerks on the right side were exaggerated. The aphasia was still very marked; the child could only say "No," "Yes," "Nurse," and this only on emotion. Deliberate voluntary speech was still absent. The heart was normal; and there was no personal or family history of rheumatism or syphilis.

The child was in good health until the onset of the hemiplegia. All, then, that could be inferred as to the origin of the attack was that it was vascular in nature. The case showed that, even in children with right-sided paralysis and aphasia due to apoplexy, a cautious prognosis should be given as to the recovery of speech.—Dr. H. WALDO said he had years ago called attention to recorded cases of motor aphasia in connection with *left* hemiplegia in left-handed people. He had never seen one of these cases, and he doubted their existence.

Dr. J. E. SHAW gave the record of "A Case of Tumour of the Crus Cerebri." A man, aged 32, was seized suddenly five months before death with slight paresis and anæsthesia of the left hand, and symptoms of defective movement of the right eye, so that from erroneous projection he could not follow his work as a shoemaker. Some weeks afterwards he was found to have complete paralysis of all the branches of the right third nerve, with a slight but variable left hemiplegia; headache and vertigo were experienced, with vomiting at rare intervals; mental hebetude deepening into occasional stupor, extension of the ocular paralysis to the superior rectus and some other muscles of the left eyeball, but not implicating the levator palpebræ, and optic neuritis three weeks before death, were developed in succession. *Post mortem* a sarcomatous tumour was found within the distended right crus cerebri, extending from nearly the optic thalamus down into the fourth ventricle, into which, through the distended aqueduct of Sylvius, the free pointed extremity of the growth penetrated.

Mr. C. A. MORTON showed a specimen of tuberculous disease of the urinary organs, from a woman aged 33, which demonstrated the beneficial results of the surgical treatment of tuberculous disease of the bladder. Six months before her death supra-pubic cystotomy had been performed, and extensive ulceration in the bladder scraped and iodoform rubbed into the ulcers; the bladder was then drained from the supra-pubic opening for three months to the great relief of the patient, but on removal of the drainage tube the old pain during micturition returned. Attacks of severe pain in the right renal region set in, and she frequently passed calculi the size of a large pin's head. After an attack of pain in this region, accompanied by vomiting which lasted for several days, she died. It was found on *post-mortem* examination that the ulceration in the bladder had completely healed, and was only represented by scar tissue; but that the left kidney was full of tuberculous abscesses, although there had been no lumbar pain on that side. The right kidney was hypertrophied, and contained only a few minute tubercles and one caseating focus; but the pelvis and calyces contained large numbers of the same kind of calculi which were passed during life, and a larger calculus was lodged in the lowest part of the ureter and could not be pressed through the orifice into the bladder. Particular attention was called to the absence of pain, tenderness, or signs of kidney enlargement in the left loin, with such extensive tuberculous disease of that kidney; and it was mentioned that Mr. Cathcart's apparatus [1] for keeping the bladder empty after supra-pubic cystotomy had been found very useful in the treatment of the case. Reference was made to the cases of Watson Cheyne [2] and Mansell Moullin [3] of cystotomy, in which the ulcers had either been scraped or cauterised and the bladder generally drained—in one of Watson Cheyne's cases for as long as six months. The results in these and about a dozen other

[1] *Brit. M. J.*, 1895, ii. 968.
[2] *Lancet*, 1895, i. 1579. [3] *Ibid.*, 1308.

cases on record were good. Such measures were not to be recommended early in the disease, but when other methods of treatment failed and the patient's sufferings were very great.

Dr. Henry Waldo read a paper on "The Treatment of Eczema," in which he said that it was always curable, and may be cured without the least fear of any injurious constitutional effects being produced. He raised the question as to washing or not, and insisted that the patient should be kept scrupulously clean upon an antiseptic plan, but that neither cold water nor soap should be used to the diseased area. He considered rest to the affected part essential, as also sealing it from the air, and that whatever application was used should contain some parasiticide. He entered into the selection of suitable applications for each case, and said that stimulating remedies, as a rule, should only be applied for an hour or so in the twenty-four hours. The patient's occupation may have to be enquired into, and his surroundings attended to, especially in relation to some plants and flowers. He believed that local treatment was much the most important, but internal diluents should be encouraged. He looked upon the administration of arsenic as usually harmful. He suggested means for combating the attending pruritus, and recommended intestinal antiseptics for cases of auto-intoxication, the one he mostly used being benzo-naphthol. He dealt fully with the question of alcoholic beverages and diet, and the effect of sea air. Moderate outdoor exercise was recommended as a prophylactic.—Dr. John Broom found the best results from attention to general details: in correcting errors or defects in general health, habits, and diet; in judiciously selecting and combining constitutional and local remedies; and in frequent ablutions with suitable medicinal soaps.—Dr. Bertram Rogers had recently treated patients locally with and without internal medication, and his results had been certainly better when some constitutional treatment was adopted. He was losing his faith in arsenic, and was using less and less every year; not because he had seen irritative effects produced by it, but because he had seen little if any good follow its use. Oleate of zinc ointment, to which Dr. Waldo had not referred, was a most useful application.—Mr. J. Hancocke Wathen spoke of the value of both constitutional and local treatment of eczema. Arsenic he rarely had occasion to use. Attention to the general health, and chylopoietic viscera was often sufficient internally. Locally, emollient and antiseptic treatment was of use—essentially it should be antiseptic.

Dr. Theodore Fisher read a paper entitled "Is uncomplicated Mitral Regurgitation from Valvular Disease Common or Serious?" with the object of showing that the generally recognised view that mitral regurgitation is commonly due to deformity of the mitral valve is erroneous. He pointed out that not mere thickening of the flaps, but actual deformity is necessary to produce a leak. Referring first to the clinical features of cases of rheumatism in which a systolic apex murmur becomes audible, he thought it unreasonable to suppose that sufficient deformity of the valve to produce incompetence can be produced within a few days of the onset of a first attack. He also pointed out that in first attacks of rheumatism a pulmonary systolic murmur is nearly as common as one heard at the apex, yet no one would suppose that the pulmonary murmur is produced by endocarditis of the pulmonary valve. Dr. Foxwell has shown that there is great probability that this murmur is due to dilatation of the right ventricle, mainly in the region of the conus arteriosus; and Dr. Fisher said that his own experience bore out this view of Dr. Foxwell, and he thought that the presence of systolic murmurs heard at the apex and over the pulmonary area in rheumatism frequently pointed to dilatation of both sides of the heart. In favour of such a view is the fact that under careful treatment not only the pulmonary but also the apex-murmur appearing during an attack of rheumatism frequently disappears after recovery. Still more conclusive, however, is the fact that examination of *post-mortem* records proves that deformity of the mitral valve leading to uncomplicated regurgitation is extremely rare. Dr. Fisher then referred to the importance of the fact that mitral regurgitation produced by rheumatism is due to dilatation of the heart and not to deformity of the valve.

J. PAUL BUSH, *Hon. Sec.*

---

### Plymouth Medical Society.

*December 18th*, 1895.

Dr. C. A. HINGSTON, President, in the Chair.

Dr. E. LAWRENCE FOX shewed specimens from a case of multiple sarcoma. The patient, a girl aged 16, suddenly became diplopic six months before death, and soon after developed lumps in both mammæ with pains across shoulders and in right knee. Her legs became weaker, until one morning she was unable to stand. Then the legs became immobile, and sensation was impaired; incontinence of urine followed, and various lymphatic glands in neck and axillæ became involved. Three months before death numerous hard nodules appeared in abdominal walls, and headache was complained of, followed by continual vomiting. A month later there was deafness, and the tongue deviated to the right. The temperature was raised throughout, sometimes to 103° or 104°, till death occurred. At the necropsy sarcoma was found generally throughout the body. The peritoneum and mesenteric glands were largely affected; both ovaries and kidneys contained numerous new growths—calcareous in the former, but cystic in the latter; one growth was found in each lobe of the liver, but none in spleen or lungs. The mammæ consisted almost entirely of new growths. The spinal cord was not affected. Brain not examined.

Mr. A. G. RIDER showed a cyst removed from the right occipital region of a girl, aged 6 years. At birth there was a swelling in this situation which became tense on crying. Latterly, mother noticed that the swelling did not vary in size as formerly. The tumour on incision yielded clear fluid; the cavity was lined with a perfectly smooth membrane, and had a small process or neck at one point. It was regarded as an obliterated lateral occipital meningocele.

---

*January 2nd*, 1896.

Dr. C. A. HINGSTON, President, in the Chair.

Mr. W. L. WOOLLCOMBE read a paper on "The Surgical Aspects of Appendicitis."

*February 8th*, 1896.

Dr. C. A. Hingston, President, in the Chair.

Mr. R. H. Lucy demonstrated the results of the radical treatment of inguinal hernia after relief of strangulation in a man, aged 66, operated on two years ago by Mitchell Banks's method. He also showed a man, aged 45, whose pile-bearing area had been removed six months ago by Whitehead's method.

Mr. W. C. Hamilton brought forward for Mr. Whipple a young man with primary sore and secondary rash, now under treatment by increasing doses of anti-syphilitic serum. He also exhibited a number of calcareous particles coughed up by a man, aged 25, with phthisical signs in both lungs.

Mr. G. F. Aldous exhibited (1) Sub-periosteal sarcoma, springing from the back of the lower end of the right femur, infiltrating tissues in the popliteal space and commencing to attack the bone. The tumour was a hemorrhagic diffluent mass, and had been growing rapidly for four months in a woman aged 48. The thigh was amputated through the upper third. (2) Fracture running through the body of the sphenoid bone, lacerating the anterior cerebral arteries, with hemorrhage into the frontal lobes. The patient, a man aged 55, of alcoholic habits, fell down eight steps at 11 p.m., got up, said he felt "all right," went to bed and became unconscious, but was treated by friends as drunk. He was seen on morning of third day, and found to be partly comatose. There was no paralysis; but he lapsed into coma, with incontinence of urine and fæces, and died on 6th day.

Dr. Webber showed a heart removed from a man, aged 54, who died from angina pectoris. It weighed fourteen ounces; the surface of the ventricle was loaded with fat, and the myocardium was fatty. Ventricles distended with *ante-mortem* clot, and the mitral valve contained one or two calcareous nodules, but was competent. The aortic valves were atheromatous and incompetent, each sinus of Valsalva being calcareous. Of the coronary arteries, the right was calcified for over an inch from its origin, and the left for rather less.

---

*February 26th*, 1896.

Dr. C. A. Hingston, President, in the Chair.

Mr. A. G. Rider read a paper on "Some Affections of the Throat."

---

*March 21st*, 1896.

Dr. C. A. Hingston, President, in the Chair.

Mr. J. Norris showed a well-marked case of pseudo-hypertrophic paralysis in the second stage in a boy aged 8 years.

Mr. C. E. Russel-Rendle showed (1) Three generations in one family, aged 10 years, 39 years, and 59 years respectively, with well-marked lamellar cataract. (2) A bony plate perforated by the central artery of the retina, found in the choroid of an eye removed for pan-ophthalmitis in a woman aged 43.

Mr. R. H. Lucy exhibited (1) An old fracture in the outer fourth of the right clavicle in a man aged 56, with downward displacement of the arm and outer fragment. (2) A haulier, aged 30, with marked prominence of each acromial end of his clavicles and lax acromio-clavicular ligaments, allowing an apparent dislocation of the latter.

Mr. W. L. Woollcombe showed (1) A finger on the dorsum of which was a growth resembling a sarcoma, but which was found on microscopical examination to be tubercular; the focus being a depression on the side of the head of the metacarpal bone. A slide, prepared by Dr. Webber, was shown with it. (2) A photograph of a man, aged 50, whose posterior ulnar ridges showed three symmetrical nodes. There was a history of two attacks of acute rheumatism, but no peri- or endocardial mischief.

Dr. Pethybridge showed (1) A slide of diphtheritic membrane with Löffler's bacilli *in situ*; and (2) A slide of bacteria of the mouth.

Mr. J. E. Square exhibited a specimen of an eye showing retinal detachment, and a plate of bone in the choroid in a male aged 55.

---

*April 11th*, 1896.

Dr. C. A. Hingston, President, in the Chair.

Dr. Charles Aldridge read a paper on "Testamentary Capacity."

R. H. Lucy, *Hon. Sec.*

---

### Swansea Medical Society.

*March 3rd*, 1896.

Dr. D. Arthur Davies in the Chair.

Dr. N. Fox Edwards showed a man, aged 38, with malignant disease of the tonsil and soft palate. When the patient first came under observation, about six weeks previously, the extent to which the growth had infiltrated the surrounding tissues negatived any operative measures, and during this six weeks he had been treated with pyoktanin, 30 minims of a saturated solution having been injected daily into the substance of the growth. Very little pain or hemorrhage was caused by the injections. During this period the growth appeared to remain in a stationary condition, though the patient himself strongly affirmed that it was decreasing.—Mr. Brook considered that the chief effect of the pyoktanin had been to render the whole malignant mass much softer; but that no real improvement had taken place.—Dr. Lancaster suggested that if the supposed action of the aniline dyes upon malignant new growths were due, as generally stated, to their selective affinity for the proliferating nuclei, the action of any particular aniline dye upon a morbid growth should stand in direct ratio to its affinity for cell nuclei: hence safranin, which of all the anilines has probably the greatest affinity for nuclei, might be expected to yield better results than pyoktanin.[1]

Mr. Kynaston Couch exhibited a tonsillar calculus, about the size of a large hazel-nut, which he had removed from the tonsil of a woman, aged 24. When fresh it weighed about 50 grains, and when dried about 30 grains. Originally this calculus must have been a very large one, as the patient stated that twelve months previously a piece of "stone" of about equal size had been removed from the same tonsil.

---

*March 17th*, 1896.

Dr. F. Knight in the Chair.

Dr. A. F. Blagdon Richards read a paper on "Pathogenic and Non-Pathogenic Micro-Organisms," contending that the characteristics of the various micro-organisms known at present are by no means fixed; but that under suitable conditions micro-organisms which ordinarily exert no specific pathogenic effect upon human tissues may develop the capacity to produce such an effect, and *vice-versâ*.

---

*April 7th*, 1896.

Mr. E. B. Evans, President, in the Chair.

Dr. R. C. Elsworth showed a specimen of an ovarian cyst with twisted pedicle. The patient from whom he had removed the cyst was a middle-aged spinster with the following history: Her general health was good, and she was ignorant of the existence of a tumour. A small fire had broken out in her room, and she extinguished it by throwing several buckets of water over it; in doing this she swung the full buckets from right to left, and so employed her abdominal muscles to an unaccustomed degree. Immediately afterwards she was seized with a violent pain in the lower abdomen and vomited. When seen by Dr. Elsworth she was in extreme pain and somewhat collapsed; and a tense rounded tumour reaching some five inches above the pubes was easily seen and felt on abdominal palpation. Ovarian cystoma with twisted pedicle was diagnosed, and ovariotomy was performed thirty hours after the symptoms first appeared. When the abdomen was opened, the tumour was found to be a cystoma of the right ovary, and one complete revolution of the tumour upon its pedicle had taken place, so that the original anterior surface of the cyst still presented anteriorly. The rotation had occurred *from right to left*, *i.e.* in the same direction as the patient had exerted unusual muscular force in swinging the full buckets. There was no actual peritonitis, but the veins on the surface of the cyst were distended with dark blood, and the pedicle and the surrounding tissues were very deeply congested and dusky-red in colour. It was necessary to place the ligature on the pedicle very close to the uterus; but the operation otherwise was uncomplicated. The woman did very well for about a day and a half,

[1] May 10th. Since the former date the growth has rapidly enlarged, broken down, and ulcerated; the daily injections of pyoktanin have been steadily persevered with. The patient has been practically free from pain both before and also during this treatment. From its appearance the growth is probably a sarcoma.

then she became suddenly collapsed and died. At the autopsy it was found that the ovarian vein had ruptured some three inches above the pedicle, causing profuse internal hemorrhage. Dr. Elsworth remarked that he had not succeeded in finding any case recorded in which death after ovariotomy had resulted through rupture of the ovarian vein. In the discussion which followed it was generally considered that the rupture was due to the morbid condition of the vein-wall, resulting from the interference with the blood circulation consequent upon the rotation.

---

*April 21st*, 1896.

Mr. E. B. Evans, President, in the Chair.

Dr. N. Fox Edwards read a paper upon "The Medicinal Treatment of Phthisis," referring especially to the good effects of a long-continued exhibition of guaiacol.

---

*May 14th*, 1896.

Mr. E. B. Evans, President, in the Chair.

Dr. Isambard Owen, Senior Deputy Chancellor of the University of Wales, and Physician to St. George's Hospital, delivered an address on "The Prevention of Phthisis."

In the evening the annual dinner was held, when thirty-nine members of the Society and visitors were present.

---

*Annual Meeting, May 19th*, 1896.

Mr. E. B. Evans, President, in the Chair.

The following officers were chosen for the ensuing year:—President; Dr. H. A. Latimer: Vice-President; Mr. E. B. Evans: Treasurer; Mr. J. Kynaston Couch: Committee; Mr. E. Davies, Dr. F. Knight, Dr. R. C. Elsworth, Dr. E. Forsyth: Honorary Secretaries; Dr. E. Le Cronier Lancaster and Mr. J. K. Couch.

E. Le Cronier Lancaster, } *Hon.*

J. Kynaston Couch, } *Secs.*

---

### Torquay Medical Society.

*April 15th*, 1896.

Dr. Reginald Pollard, President, in the Chair.

The President read notes of "A Case of Cæsarean Section," performed by him on a woman who was sent into the hospital in labour; but where delivery in the ordinary way was prevented by a cysto-fibroma springing from the lower part of the back of the uterus. The woman had been some time under chloroform before the operation, whilst attempts were made to deliver; but it was found impossible, and Cæsarean section was performed. The uterine wound was closed by superficial and deep sutures of carbolised silk, inserted by the Sänger-Leopold method. It was not thought advisable to attempt removal of the uterus and tumour, which had numerous adhesions to the pelvis. The patient unfortunately never rallied, and died from shock within forty-eight hours. P.M.E.: Abdomen distended with flatus; but no sign whatever of peritonitis. Uterine wound and abdominal peritoneum united and healthy. The tumour, which was larger than a child's head, was connected by a pedicle to the posterior part of the uterus or upper part of cervix—there were numerous adhesions to the pelvis. There was general thickening of the uterine walls, and one or two other fibroids commencing. An incision into the tumour showed that it was cystic and contained a yellowish fluid. The patient, whose menstrual flow was slightly excessive, had been married six years, and had had three miscarriages and one six-months' premature birth; but no tumour had been discovered until she was in labour and was seen by Drs. Andrew and Winter.

Mr. WINTER read a paper on "Diphtheria."

G. YOUNG EALES, *Hon. Sec.*

